# Modulation of mGlu2 Receptors, but Not PDE10A Inhibition Normalizes Pharmacologically-Induced Deviance in Auditory Evoked Potentials and Oscillations in Conscious Rats

**DOI:** 10.1371/journal.pone.0147365

**Published:** 2016-01-25

**Authors:** Abdallah Ahnaou, Ria Biermans, Wilhelmus H. Drinkenburg

**Affiliations:** Department of Neuroscience, Janssen Research & Development, A Division of Janssen Pharmaceutica N.V., Turnhoutseweg 30, B-2340 Beerse, Belgium; Hudson Institute, AUSTRALIA

## Abstract

Improvement of cognitive impairments represents a high medical need in the development of new antipsychotics. Aberrant EEG gamma oscillations and reductions in the P1/N1 complex peak amplitude of the auditory evoked potential (AEP) are neurophysiological biomarkers for schizophrenia that indicate disruption in sensory information processing. Inhibition of phosphodiesterase (i.e. PDE10A) and activation of metabotropic glutamate receptor (mGluR2) signaling are believed to provide antipsychotic efficacy in schizophrenia, but it is unclear whether this occurs with cognition-enhancing potential. The present study used the auditory paired click paradigm in passive awake Sprague Dawley rats to 1) model disruption of AEP waveforms and oscillations as observed in schizophrenia by peripheral administration of amphetamine and the N-methyl-D-aspartate (NMDA) antagonist phencyclidine (PCP); 2) confirm the potential of the antipsychotics risperidone and olanzapine to attenuate these disruptions; 3) evaluate the potential of mGluR2 agonist LY404039 and PDE10 inhibitor PQ-10 to improve AEP deficits in both the amphetamine and PCP models. PCP and amphetamine disrupted auditory information processing to the first click, associated with suppression of the P1/N1 complex peak amplitude, and increased cortical gamma oscillations. Risperidone and olanzapine normalized PCP and amphetamine-induced abnormalities in AEP waveforms and aberrant gamma/alpha oscillations, respectively. LY404039 increased P1/N1 complex peak amplitudes and potently attenuated the disruptive effects of both PCP and amphetamine on AEPs amplitudes and oscillations. However, PQ-10 failed to show such effect in either models. These outcomes indicate that modulation of the mGluR2 results in effective restoration of abnormalities in AEP components in two widely used animal models of psychosis, whereas PDE10A inhibition does not.

## Introduction

Deficits in sensory information processing have been associated with several neuropsychiatric and neurodegenerative disorders. Investigation of the electrophysiological response to visual and auditory stimuli may be used to elucidate the neurobiological processes underlying such diseases as well as to evaluate treatments [1,2]. Much effort in recent translational psychiatric research has focused on using neurophysiological measurements to characterize endophenotypes of schizophrenia and neurobiological markers underlying the disease. Schizophrenic patients exhibit disturbances in inhibitory filtering mechanisms that are considered a trait marker for the illness [3,4]. Event related potentials (ERPs) are widely used to study disruption of neuronal circuits underlying sensory encoding, information processing and attention in neuropsychiatric and neurodegenerative disorders. The auditory paired-stimulus P50 paradigm is a predominant neurophysiological tool used to demonstrate deficiency in gating or filtering out stimuli that lack novelty, threat or other salience. An animal analogue of the human P50 AEP has been developed to investigate the underlying neural integrity of inhibitory circuits and to explore potential abnormalities in the mechanisms of the P50 suppression found in schizophrenia [1,2]. Sensory gating, a process by which the response of the brain to a repeated stimulus is attenuated, contributes to information processing by enabling organisms to filter extraneous sensory inputs from the environment [5,6]. Changes in the amplitudes of AEP responses in humans and animals can be demonstrated in the double click paradigm, in which two identical auditory tones are presented in a time window of 500 ms. Normal subjects have a smaller response to the second stimuli tone (S2) compared to the first stimuli tone (S1), and the ratio measure (S2/S1) is used as a quantitative index of sensory gating. The P1 component of the AEPs reflects largely pre-attentive sensory processing, while N1 and P2 reflect attentional processing (relative to the late endogenous P300 component involving voluntary, controlled attention). Disturbances in information processing and cognitive function are key features of schizophrenia. Accordingly, abnormalities in P50 suppression have been related to deficits in attention and processing speed assessed in cognitive tests [7,8–12]. In schizophrenia, deficits in P50 gating may result from deficient response to first, second or both stimuli. The first condition referred as “gating out” is assoiated with deficient suppression of the response to the second stimuli with fairly normal amplitude of S1 wave, which is assumed to reflect failure and diminished capacity of the brain to inhibit repetitive irrelevant sensory input [13,14]. The second condition referred as “gating in” is observed when a relatively small amplitude S1 wave occurrs with a fairly normal amplitude S2 wave [14–17], which reflects an inability of the brain to encode and register novel fetaures of first stimuli. These waveform properties of waveforms measured in the paired click paradigm may correlate with the ability of the brain to “gate out” by decreasing the S2 response to repetitive irrelevant stimuli and to “gate in” by increasing the S1 response to novel or changing stimuli [18]. Schizophrenic patients commonly exhibit deficits in sensory gating in double click paradigms, which can be replicated in animal models of schizophrenia wherein suppression of the response to the second stimulus is reduced [3,4,6,19–22].

Recently, analyses of brain electroencephalogram (EEG) network oscillations have also gained support as a sensitive measure of both normal, abnormal information processing and cognitive functions in humans as well as in animals. Oscillations reflect synchronization in the activity of neuronal ensembles that arise either spontaneously or in response to an event, such as an auditory stimulus. Oscillatory activities are estimated by performing a decomposition of the EEG signal into phase and magnitude information over a range of frequencies that have been correlated with various states of perceptual and cognitive processing. Network oscillations in the gamma range have repeatedly been shown to accompany several distributed neurocognitive functions in normal subjects [23,24]. A wealth of evidence indicates that abnormalities in the oscillatory activity of neuronal networks in the gamma frequency range play a central role in the pathophysiology of schizophrenia [25–31]: pathologically increased gamma oscillation propensity is found during psychotic episodes and hallucinatory states [27,28,32–34]. In animals, the NMDA receptor antagonists PCP, ketamine and MK801 elicited aberrant gamma oscillations in the hippocampus and neocortex,which likely reflects similar cortical EEG abnormalities found in schizophrenics [35–38]. Thus, the ability to induce and maintain large network oscillations within the gamma frequency range in rat offers the possibility of linking the results of clinical studies to findings from basic research.

Most clinically effective atypical antipsychotics show mixed dopamine (D2) and 5-hydroxytryptamine (5-HT_2A_) antagonism, resulting in an improved safety profile regarding extrapyramidal symptoms and increased antipsychotic efficacy against psychosis [39]. However, the resistance of negative and cognitive symptoms to these treatments supports the hypothesis that other systems are involved in the pathophysiology of schizophrenia. On the one hand, the disruption of intracellular signaling pathways involving cAMP and/or cGMP as second messengers plays a key role in the disease. Inhibition of the cyclic nucleotide phosphodiesterase (PDE) 10A has been recently hypothesized to represent a new therapeutic approach for treating schizophrenia [40]. PDE10A is highly expressed in the medium spiny neurons of the mammalian striatum [41,42], where this enzyme regulates both cAMP and/or cGMP signaling cascades and striatal output to the cortico-striato-thalamic and nucleus accumbens circuits [43].

On the other hand, pharmacological, anatomical and genetic studies indicate that deficits in glutamatergic neurotransmission contribute to all symptoms of schizophrenia [44,45]. Lower levels of glutamate have been observed in the cerebrospinal fluid and postmortem brain tissue of schizophrenic patients. In addition, the non-competitive N-methyl-D-aspartate (NMDA) receptor antagonists phencyclidine (PCP) and ketamine have been shown to elicit transient psychosis, disrupted mood and cognitive process in healthy volunteers [45,46], which could be attenuated by administration of antipsychotics [47]. Moreover, modulation of the glutamatergic neurotransmission using an orthosteric agonist of the metabotropic glutamate receptor (mGluR2) demonstrated antipsychotic-like effects in a variety of preclinical in vivo paradigms [7,48,49]. Furthermore, an mGluR2 prodrug (LY2140023) showed efficacy and reduced symptoms in schizophrenic patients [50].

In the present study, we have used PCP and amphetamine in the paired click paradigm in conscious rats i) to model disturbances in the AEP waveform components observed in schizophrenia and ii) to evaluate the degree to which the mGluR2 agonist LY404039 [48] and the PDE10 inhibitor PQ-10 [51] could improve PCP and amphetamine-induced auditory processing deficits as was observed with atypical antipsychotics risperidone and olanzapine, respectively.

## Material and Methods

### 1. Animal husbandry

All protocols were performed in accordance with guidelines of the Association for Assessment and Accreditation of Laboratory Animal Care International (AAALAC), and of the European Communities Council Directive of 24 November 1986 (86/609/EEC) and were approved by Janssen Pharmaceutica ethical committee. All animal studies have been carried out in accordance with guidelines of the Association for Assessment and Accreditation of Laboratory Animal Care International (AAALAC), and of the European Communities Council Directive of 24 November 1986 (86/609/EEC) and were approved by Janssen Pharmaceutica Ethical Committee. Every effort was made to minimize animal use and disturbances in animal well-being and experimental animals were euthanized at the end of the study by common rodents CO2 procedure. The experiments were carried out on male, adult Sprague Dawley rats, supplied by Harlan Netherlands, weighing 400–560 g at the time of experiments. Ninety-six animals were housed in individually ventilated cages (IVC), located in a sound-attenuated chamber. The rats were provided with micro-chips for identification purposes and maintained under controlled environmental conditions throughout the study: 22°C ± 2°C ambient temperature, relative humidity 60%, 12:12 light-dark cycle (lights off from 07:00 to 19:00; light intensity: ~100 lux). Standard laboratory food chow and tap water were available ad libitum. Experiments were conducted during the dark phase of the circadian time between 9 am and 1 pm under a reversed light-dark schedule.

### 2. Surgery and experimental design

The surgery was performed under isoflurane inhalation anesthesia. A mixture of 30% O2, 70% N2O and 5% isoflurane was administered to animals as an initial induction for 2 minutes. Then, the animals were mounted in a stereotaxic apparatus and were given a continuous constant mixture of O2, N2O and 2% isoflurane. An analgesic Piritramide (dipidolor) 0.025 mg/kg (0.1 ml / 100 g. B.W) was subcutaneously administered before an incision was made over the total length of the head. The oval area of the scalp was removed and the uncovered skull was cleared of the periosteum. Animals were equipped with fixing epidural screws for EEG recordings (diameter 1 mm), which were placed bilaterally on the surface of the left and right hemisphere along the antero-posterior axes (frontal left: FL, frontal right: FR, parietal left: PL, parietal right: PR, occipital left: OL, occipital right). Epidural electrodes were stereotaxically fixed at the following coordinates (AP +2 mm, L +/- 2 mm; AP -3 mm, L +/-4 mm and AP -5.5 mm, L +/- 4 mm from the Bregma, respectively) and referenced to the same ground electrode placed on the midline above the cerebellum. The incisor bar was -5 mm under the center of the ear bar, according to the stereotactic atlas of Paxinos and Watson [52]. Screw electrodes were soldered to insulated wires (7N51465T5TLT, 51/46 Teflon Bilaney, Germany) connected to a pin (Future Electronics: 0672-2-15-15-30-27-10-0) with a small insert (track pins; Dataflex: TRP-1558-0000) and were fit into a 10-hole connector after which the whole assembly was affixed with dental cement to the cranium.

### 3. Paired-stimulus auditory evoked potentials: recording and analysis

Two weeks after surgery, the animals were habituated to the recording procedure by connecting them at regular intervals to a rotating swivel, allowing free movement while EEG and evoked responses were monitored in the paired-stimulus paradigm.

The P50 paradigm consists of two consecutive auditory clicks presented 500 ms apart (S1: first click or conditioning stimulus, S2: second click or test stimulus). The P50 gating is the relative amplitude reduction of auditory evoked potential P50 from the first stimulus (S1) to the second stimulus (S2), which mostly has been reported as a ratio measure (S2/S1).

Individual animals were kept in their home cage placed in recording Faraday boxes for at least 15 min before the start of the recording session in order to reduce any kind of stress. The timing of waveform averages was determined on the basis of 1) our balanced stimulus presentation protocol and 2) the compounds’ pharmacokinetics, while minimizing confounding effects of stimulant-induced stereotyped motoric hyperactivity on the one side and response habituation due to repeated stimulus presentations on the other side: @1) A 30 min stable EEG baseline session without stimuli was used to check the quality of signals, after which a subsequent drug-naive baseline session of 30 min followed with stimuli (120 trials: 3 runs of 40 trials “7 min each” with a 3 min white noise between trials): hence the baseline period in total lasted 2 times 30 min = 60 min. This series of 120 clicks (3000 Hz tones and 10-ms duration) was presented in pairs of 500 msec at 10s inter-stimulus interval at 87 dB compared with background of 40 dB. In the next phase of the experiment, a 120 min total session followed during which the challenge drugs were administered in the combined pharmacological treatment: this session included 4 series of 120 trials (each series lasted 30 min, so 4 times 30 = 120 min, 480 trials). In this second series of trials, the last 2 runs (i.e. trials 241–280) because of of habituation due to repeated stimulus presentation, only stimulus presentations with low levels of motor behavior and artifacts-free epochs were selected only to construct waveforms and calculate average peak amplitude for different AEP components. @2) Given the PK-characteristics of the drugs used (e.g. stereotyped behavior of challenge stimulant drugs will wear off after about 30 minutes, while PK indicates t ½ between 60–120 minutes; test compounds t ½ about 2–3 hrs), Therefore the waveform averages were analyzed while both compound PK characteristics were adequate, yet confounding effects of stereotyped behavior or stimulus habituation minimal.Stimuli were generated by sound hardware and software (Labview Electronic) and were delivered through speakers mounted at the cage top at approximate height of 20 cm to allow relatively uniform intensity distribution in the cage.

Offline, the AEP waveforms were filtered between 1 and 500 Hz and baseline corrected at stimulus onset. In all animals, the AEP waveforms were chosen from the the frontal electrode sites, the areas showing pronounced auditory potential response, were consequently used for all subsequent pharmacological studies. Waveform averages for each rat were calculated at 40–60 min after the administration of challenge drugs on epochs that were free of gross movement artefacts; sleep state and amplifiers saturation artifacts. Individual sweeps were rejected for movement artifacts based on a criterion of 2 times the root mean squared amplitude per rat. Grand average waveforms were constructed in 50 ms pre-stimulus and a 450 ms post-stimulus interval for each treatment. The resultant average for each subject were analyzed for P1, N1 and P2 component amplitudes.

In all animals, the changes in frequency oscillations were analyzed by using the Discrete Fourier Transform on passive wake epochs used to construct AEP waveforms. For baseline EEG power, spectral density values were averaged for both S1 and S2 responses in the runs of baseline session with stimuli. For drug effects on EEG oscillations, EEG spectral power was averaged for both S1 and S2 responses in selected passive waking epochs between 40–60 min after the administration of challenge drugs. Drug-induced changes in EEG spectral power during S1 and S2 were calculated as the ratio of mean spectral power obtained following the administration of drug in S1 and S2 responses versus the mean spectral power obtained during baseline with stimuli period in S1 and S2, respectively. This procedure allows for assessment of drug-induced changes in EEG power of S1 and S2 responses expressed in each frequency bands: Delta band (1–4 Hz), Theta band (theta1: 4–6.5 Hz, theta2: 6.5–8 Hz), Alpha band (alpha1: 8–11 Hz; alpha2: 11–14 Hz), Beta band (beta1: 14–18 Hz; beta2: 18–32 Hz), Gamma band (gamma1: 32–48 Hz; gamma2: 52–100 Hz).

### 4. Auditory evoked potentials: treatment groups drugs

Treatment groups consisted of challenge drugs such as amphetamine (0.64 mg/kg) and PCP (3 mg/kg). The test drugs were risperidone (0.64 mg/kg), olanzapine (2.5 mg/kg), LY403939 (10 mg/kg) and PQ-10 (3 mg/kg). The doses selected for amphetamine and PCP were repeatedly found to elicit marked alterations in EEG patterns. The doses of risperidone and olanzapine were selected based on dopamine D2 receptor occupancy and their potency to attenuate the challenge-induced hyperlocomotor behaviour. The doses of LY403939 and PQ-10 were selected based on initial dose response AEP studies (1, 3, 10 mg/kg) and (0.3, 1, 3 mg/kg), respectively.

In the first experiments, it was examined whether subcutaneous PCP (3 mg/kg) and amphetamine (0.64 mg/kg) administration in conscious rats could induce abnormalities in the AEP components and EEG frequency oscillations similr to those described in schizophrenic patients. Subsequently, it was determined to what extent the atypical antipsychotics risperidone (0.64 mg/kg) and olanzapine (2.5 mg/kg) would normalize PCP and amphetamine-induced aberrant AEP waveform components and EEG oscillations, respectively. In the second set of experiments, it was evaluated whether oral administration of LY403939 (10 mg/kg) or PQ-10 (3 mg/kg) had therapeutic potential across the NMDA receptor antagonist and dopamine agonist models of schizophrenia.

Animals were not used in equal numbers and baseline AEP responses were examined before the pharmacological experiments in order the examine the quality of EEG signal and the S2/S1 ratio measures. For each study, animals were randomly allocated to experimental conditions (n = 32 for each study, 8 animals per group). Consequently, some animals may have been used more than others (exclusion criteria: maximally 6 times reuse, and only following a wash out period of at least 2 weeks in order to avoid long-lasting effects as well as carry-over of drug treatments). Animals with an S2/S1 ratio higher than 5 indicating a disruption in gating were discarded from the analysis.

### 5. Drugs

All drugs were synthesized at Janssen Research and Development laboratories and were prepared and administered as follow: Risperidone and olanzapine (H2O + 2H2T + NaCl, subcutaneous), PCP and amphetamine (H2O + NaCl, subcutaneous). The mGluR2/3 agonist, (-)-(1R, 4S,5S,6S)-4-amino-2-sulfonylbicyclo[3.1.0] hexane-4,6-dicarboxylic acid (LY404039) (Rorick-Kehn et al., 2007) was prepared in H2O + 1NaOH for oral administration. PQ-10 (Siuciak, 2008) was dissolved in a solution of 10% Hydroxyl-propyl-beta-cyclodextrin (HP-β-CD) containing 2 equivalents of tartaric acid for oral administration. All drugs were given at a volume of 5 ml/kg of body weight in rats. An equivalent volume of vehicle was administered in control animals.

### 6. Statistical analysis

Artifact-free trials were averaged and frontal AEP waveform components such as the amplitude (expressed in μV) and latency (expressed in ms) of P1 (first most positive deflection), N1 (most negative deflection) and P2 (second most positive deflection) were analyzed. The influence of acute administration of these drugs on each auditory component, gating ratios (S2/S1) and EEG oscillations was calculated for each time point and were given as mean values ± S.E.M. Repeated measure ANOVA was applied to determine difference in relative changes for different parameters in relation to treatment. A Dunnett-Test post hoc analysis with pairwise comparison was performed to determine statistical at a significance level of p < 0.05.

## Results

### 1. Auditory gating paradigm

**Fig 1:**
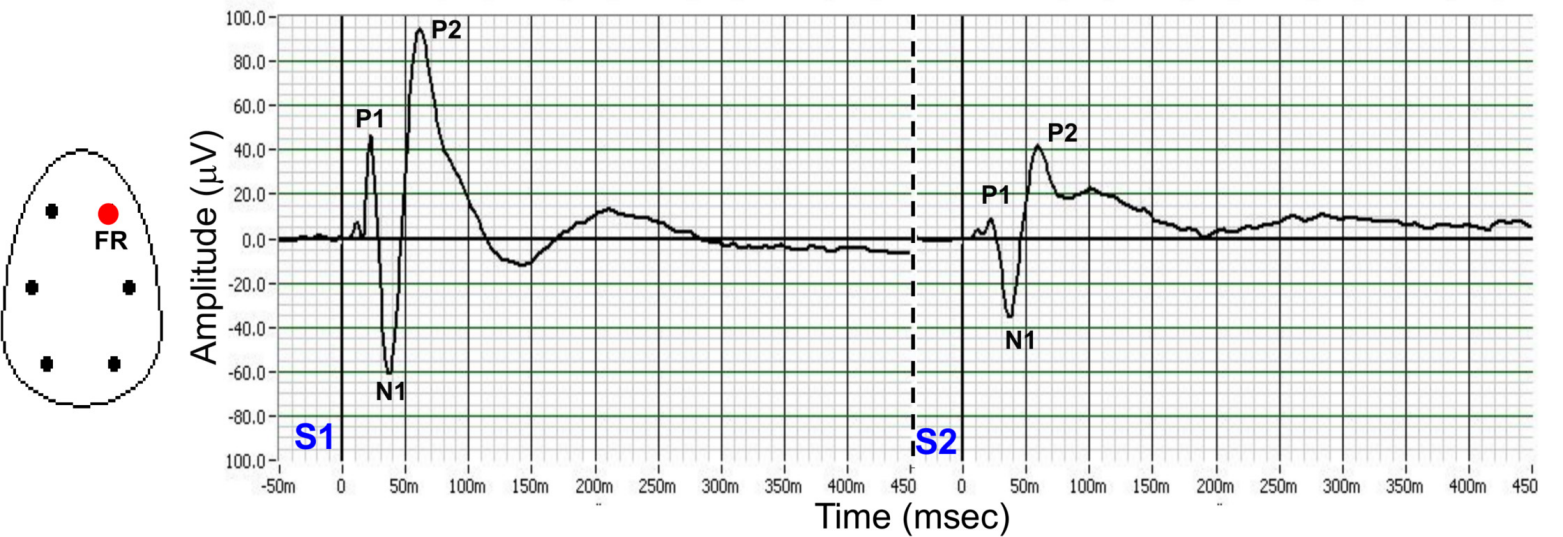
Typical grand average waveforms derived from the right frontal cortex. **Auditory-evoked responses consisted of positive and negative peak deflections (P1, N1 and P2 in response to first and second stimuli tones in awake quiet state**. A pronounced reduction in the amplitude of the second stimuli (S2) relative the first stimuli (S1) demonstrated gating for the P1, N1 and P2 waves.

Amphetamine and PCP are widely used to recreate both positive and negative symptoms of schizophrenia in rodents. Behavioral abnormalities such as hyperlocomotion and stereotypic behaviors (sniffing, head swaying, rearing) were particularly observed during the first half hour following the administration of amphetamine and PCP, therefore passive waking epochs in the interval of 40–60 min following the administration were considered here. In different experiments, typical average waveforms and peak amplitudes in response to auditory stimuli exhibited an initial positive peak P1 within 10 and 30 ms following tone stimuli, a prominent negative peak (N1) within 30 and 50 ms of stimulus delivery followed by a second prominent positive peak (P2) within 50 and 100 ms (Fig 1). A consistent reduction of the peaks P1, N1 and P2 in the response to the second stimuli compared to the first one indicated that P1, N1 and P2 exhibited gating of S2 relative to S1.

### 2. Effects of olanzapine on amphetamine-induced deviance in AEP waveform components and oscillations

#### Evoked potentials and peak amplitudes

**Fig 2:**
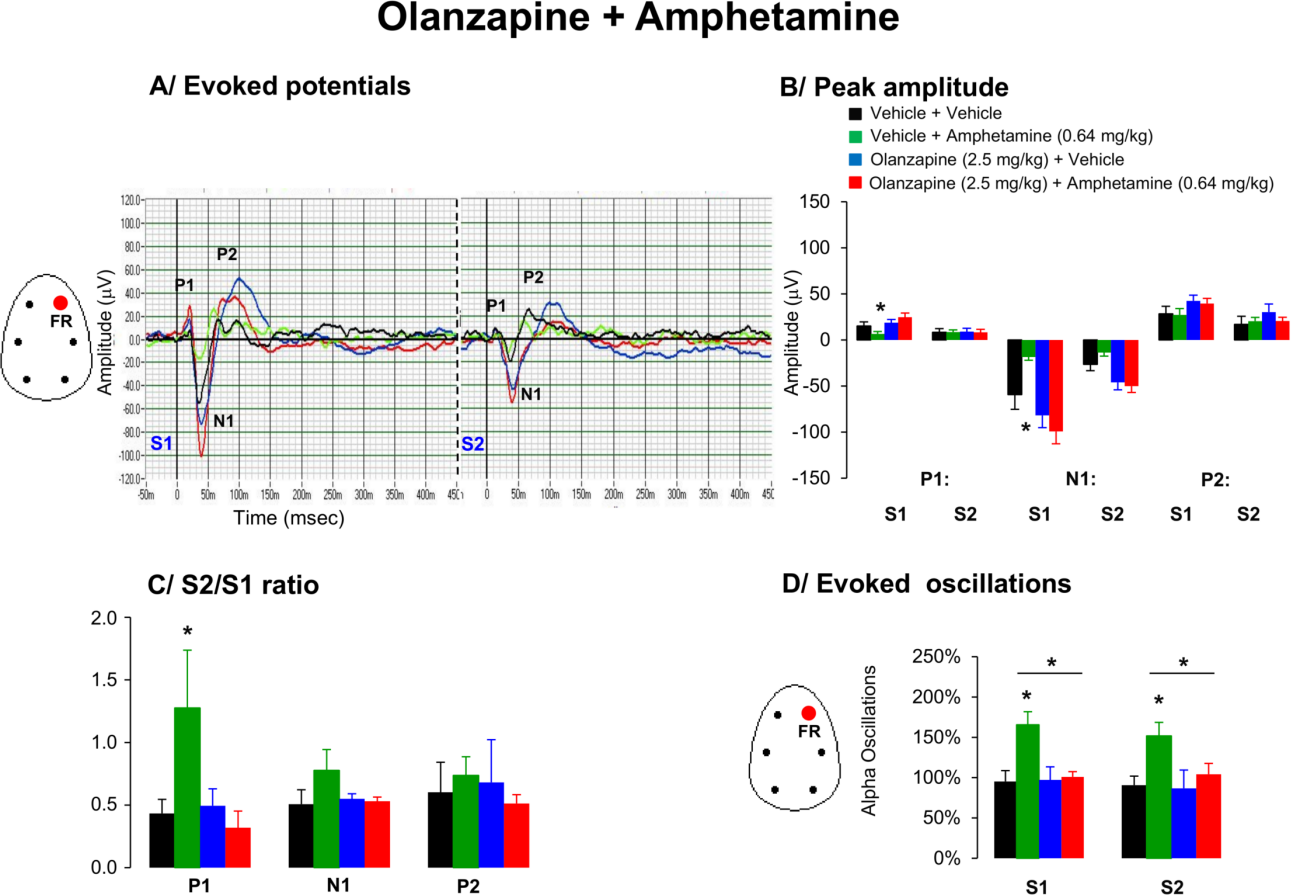
Effects of acute administration of olanzapine (2.5 mg/kg), amphetamine (0.64 mg/kg) or in combination on A/ Grand average evoked potentials derived from the frontal right hemisphere, B/ peak amplitude of the P1, N1, and P2 components (expressed in μV), C/ Mean S2/S1 ratio represent the gating index, D/ Average time frequency response in alpha EEG oscillations from the right frontal cortex in awake motionless state during the period of 40–60 min following the combined pharmacological treatment. Data are presented as mean ± S.E.M. of (n) animals for each condition (vehicle (7), olanzapine (7), amphetamine (7) and alanzapine + amphetamine (7)). 4 animals with baseline S2/S1 ratio higher than 5 across different conditions were discarded from the analysis. Olanzapine restored amphetamine-induced deficits in P1 and N1 gating responses and changes in low alpha oscillations. * indicates significant difference from vehicle (p < 0.05).

A striking difference in the morphology of the waveforms was apparent between groups for the P1, N1 and P2 waveforms components (Fig 2A). Amplitude responses of P1 and N1 to first stimuli were consistently affected by drug treatment [F(3,21) = 3.0, p = 0.03] and [F(3,21) = 10.9, p < 0.0004]. Post hoc analysis for the main effect of drug treatments revealed that amphetamine significantly decreased P1 and N1 peak amplitudes, whereas olanzapine increased N2 peak amplitude to the second stimuli (Fig 2B).

#### S2/S1 ratio

The S2/S1 ratios for both P1 and N1 amplitudes were affected by drug treatment [F(3,21) = 3.06, p = 0.04] and [F(3,21) = 2.5, p = 0.08], respectively. Post hoc tests revealed that amphetamine significantly reduced P1 and N1 ratio indices. The increased S2/S1 ratios in amphetamine-treated animals were due to reduced responses to the first stimulus rather than increased amplitude to the second stimulus. Pretreatment with olanzapine attenuated the reduced response to first stimuli therefore prevented amphetamine-induced increases in S2/S1 ratios in the frontal cortex (Fig 2C).

#### EEG oscillations

Amphetamine consistently enhanced aberrant slow alpha oscillations in both S1 and S2 responses. Pretreatment with olanzapine consistently prevented amphetamine-induced aberrant slow alpha oscillations in responses to both stimuli (Fig 2D).

### 3. Effects of risperidone and PCP on AEP waveform components and oscillations

#### Evoked potentials and peak amplitudes

**Fig 3:**
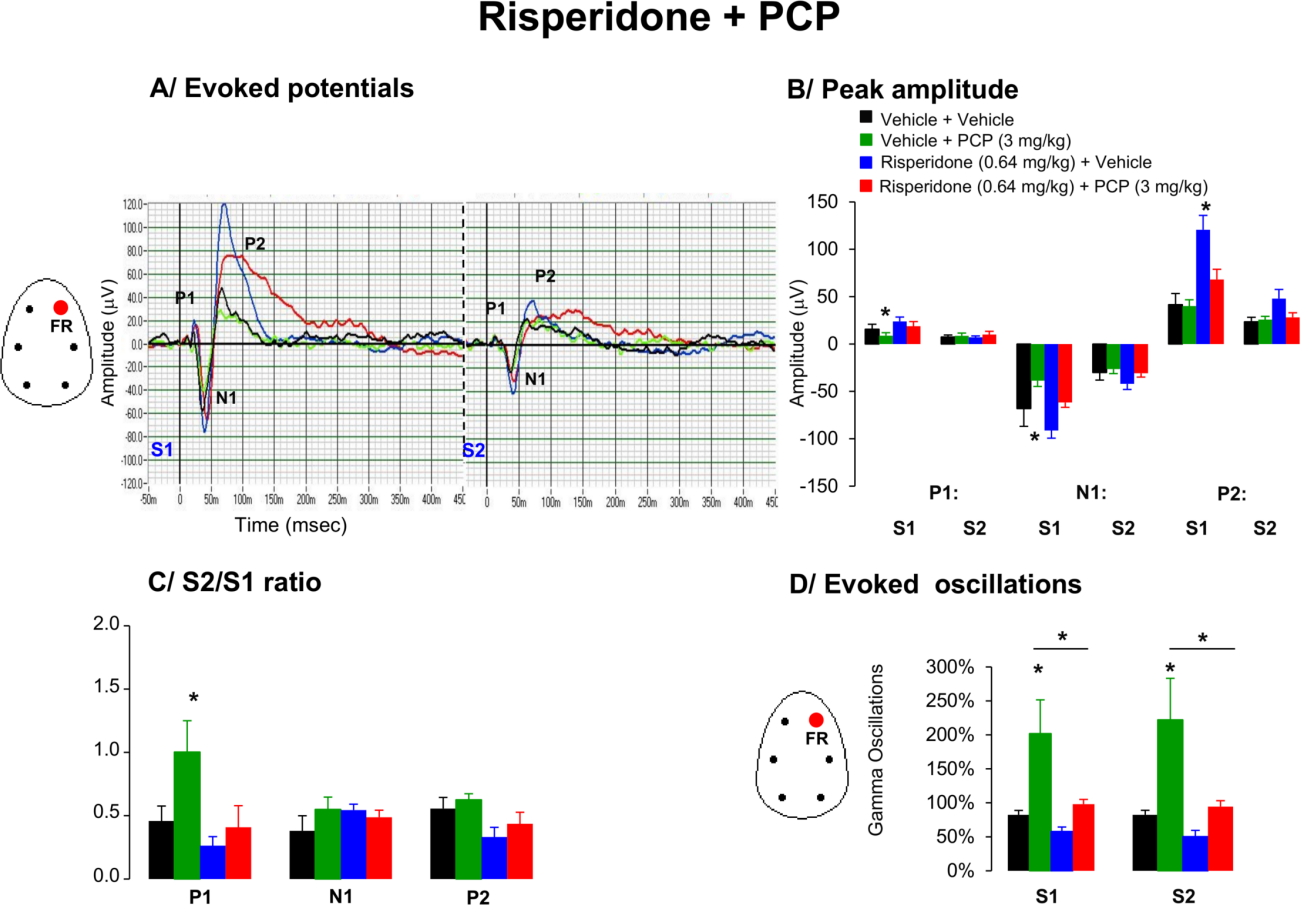
Effects of acute administration of risperidone (0.64 mg/kg), PCP (3 mg/kg) or in combination on A/ Grand average evoked potentials derived from frontal right hemisphere, B/ peak amplitude of the P1, N1, and P2 components (expressed in μV), C/ Mean S2/S1 ratio represent the gating index, D/ Average time frequency response in gamma EEG oscillations from the right frontal cortex in awake motionless state during the period of 40–60 min following the combined pharmacological treatment. Data are presented as mean ± S.E.M. of (n) animals for each condition (vehicle (7), risperidone (7), PCP (6) and risperidone + PCP (8)). 4 animals with baseline S2/S1 ratio higher than 5 across different conditions were discarded from the analysis. Administration of risperidone improved PCP-induced disruption in P1 gating response. * indicates significant difference from vehicle (p < 0.05).

The grand average waveforms of the auditory evoked potentials demonstrated a reduction of peak amplitudes to first stimuli for P1 and N1 in response to PCP (Fig 3A). The amplitude of the P1 to the first stimulus was significantly affected by drug treatment [F(3,22) = 3.05, p = 0.036] up to similar level of the corresponding peak to the second stimulus whereas the amplitude of the N1 component was close to significance level [(F3,22) = 2.8, p = 0.08]. Post hoc tests indicate that PCP markedly reduced P1 and N1 peak amplitudes (Fig 3B).

The peak amplitudes for the P1 and N1 components in response to the first and second stimulus following the administration of risperidone were not consistently different from vehicle treated group. However, risperidone increased P2 peak amplitude to the first stimulus [F(3.22) = 7.0, p = 0.007] (Fig 3B).

#### S2/S1 ratio

The S2/S1 ratio measure was consistently affected by drug treatment [F(3,22) = 3.8, p = 0.02]. Post hoc test revealed that PCP disrupted S2/S1 ratio for P1 amplitude. The disruption of the S2/S1 ratios following the administration of PCP was due to the decrease in peak amplitude to first stimuli rather than the increase peak amplitudes to second stimuli (Fig 3C). Pretreatment with risperidone effectively attenuated this PCP-induced disruption in the S2/S1 ratio for P1.

#### EEG oscillations

PCP markedly enhanced aberrant high gamma oscillations in both S1 and S2 response. Pretreatment with risperidone consistently attenuated PCP-induced aberrant high gamma oscillations in responses to both stimuli (Fig 3D).

### 4. Effects of PQ-10 and LY404039 on the P1, N1 and P2 S2/S1 ratios

#### Peak amplitudes

**Fig 4:**
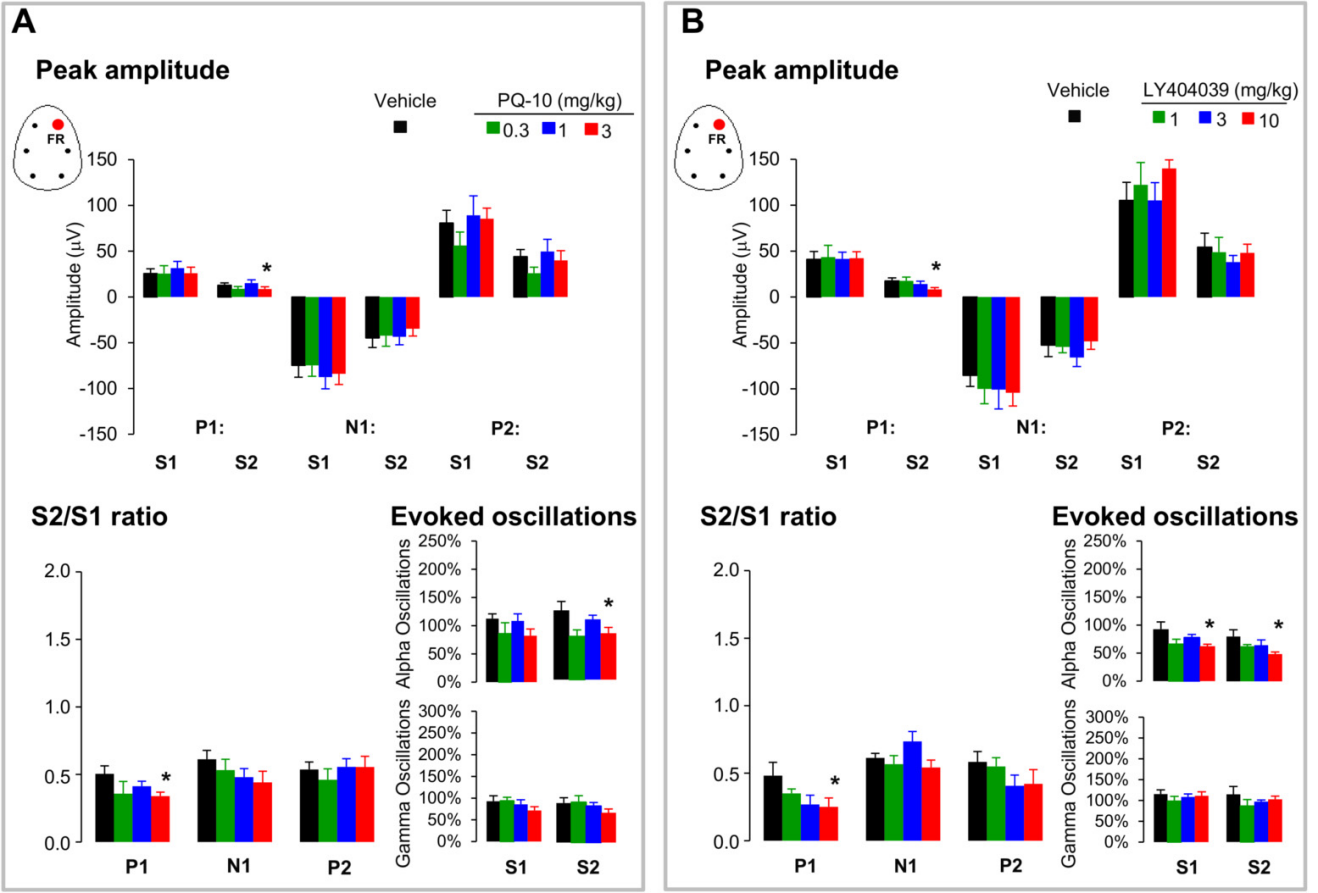
Bar graphs depicting peak amplitude of the P1, N1, and P2 components (expressed in μV) and S2/S1 ratios of frontal auditory potentials after acute administration of A/ PQ-10 and B/ LY404039 compared to vehicle values. Data are presented as mean ± S.E.M. of (n) animals for each condition: PQ10 (vehicle (7), 0.3 (7), 1 (8) and 3mg/kg (8)), and LY404039 (vehicle (6), 1 (7), 3 (7) and 10 mg/kg (8)). Animals with baseline S2/S1 ratio higher than 5 across different conditions were discarded from the analysis (2 and 3 animals in PQ-10 and LY404039 experiments, respectively). There was a significant reduction of peak amplitude and S2/S1 ratios for P1 at the highest dose of both drugs (* p<0.05).

The amplitude of the P1 was affected by drug treatment [F(3,26) = 3.7, p = 0.04]. Post hoc analysis revealed that PQ-10 at the higher dose significantly decreased P1 peak amplitudes to the second stimulus. The peak amplitudes for both N1 and P2 to the first stimulus were enhanced, however these changes did not reach significance level (Fig 4A).

#### S2/S1 ratio

The S2/S1 ratio for the P1 amplitude was influenced by drug treatment [F(3,26) = 2.9, p = 0.07]. Post-hoc analysis showed that PQ-10 at the highest dose significantly reduced the S2/S1 p = 0.043 (Fig 4A). The S2/S1 ratio for N1 and P2 amplitudes were not consistently affected by drug treatment [F(3,26) = 2.05, p = 0.14] and the P2 amplitude [F(3,26) = 2.22, p = 0.17].

#### EEG oscillations.

PQ10 at the higher dose slightly decreased EEG slow alpha and higher gamma oscillations in response to both S1 and S2 auditory stimuli.

#### Peak amplitudes

The amplitude of P1 amplitude was affected by drug treatment [F(3,25) = 4.1, p = 0.04]. Post hoc analysis showed that LY404039 at the highest dose consistently decreased P1 amplitude to the second stimulus. Both N1 and P2 peak amplitudes were enhanced, however these changes did not reach significance level.

#### S2/S1 ratio

The S2/S1 ratio for the P1 amplitude was affected by drug treatment [F(3,25) = 3.56, p = 0.046]. Post hoc analysis revealed that LY404039 at the highest dose consistently lowered the S2/S1 ratio effect of p = 0.04 (Fig 4B). There were no effects of LY404039 on S2/S1 ratios for the N1 amplitude [F(3,25) = 0.26, p = 1.03] and the P2 amplitude [F(3,25) = 0.40, p = 0.8].

#### EEG oscillations

LY404039 at the higher dose consistently decreased aberrant EEG slow alpha in responses to both S1 and S2 auditory stimuli. A slight decrease in higher gamma oscillations was also found.

Next, the higher doses of PQ-10 and LY404039 previously shown to decrease S2/S1 ratio for the P1 amplitude were used in separate combined pharmacological experiments with amphetamine and PCP.

### 5. Effects of PQ-10 and amphetamine on AEP waveform components and oscillations

#### Evoked potentials and peak amplitudes

**Fig 5:**
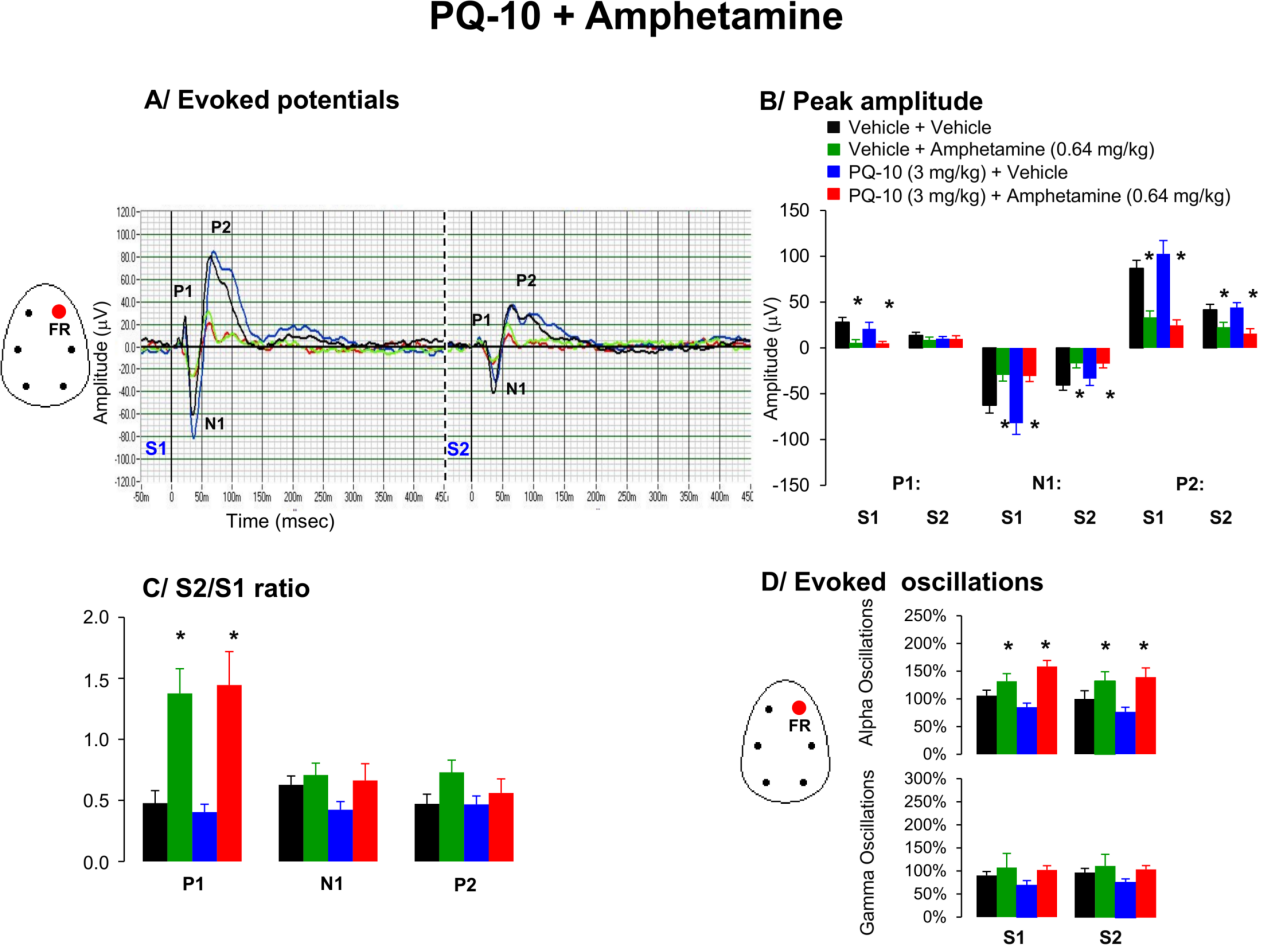
Effects of acute administration of PQ-10 (10 mg/kg), amphetamine (0.64 mg/kg) or in combination on A/ Grand average evoked potentials derived from frontal right hemisphere, B/ peak amplitude of the P1, N1, and P2 components (expressed in μV), C/ Mean S2/S1 ratio represent the gating index, D/ Bar graphs indicate average time frequency response in alpha and gamma EEG frequency oscillations from the right frontal cortex in awake motionless state for the period of 50–60 min following the second pharmacological treatment. Data are presented as mean ± S.E.M. of (n) animals for each condition (vehicle (7), PQ10 (7), amphetamine (8) and PQ10 + amphetamine (8)). 2 animals across different conditions with baseline S2/S1 ratio higher than 5 were discarded from the analysis. Administration of PQ10 failed to improve amphetamine-induced disruption in gating response. * indicates significant difference from vehicle (p < 0.05).

Grand averages of the auditory evoked potentials show that amplitudes of P1, N1 and P2 were affected by drug treatment: P1 [F(3.25) = 5.4, p = 0.008], N1 [F(3.25) = 10.4, p = 0.00001] and P2 [F(3.25) = 15.6, p = 0.00001]. Post hoc tests revealed that amphetamine significantly reduced P1 and N1 peak amplitudes, whereas PQ-10 (3 mg/kg) had no consistent influence on the amplitude of the P1, N1 and P2 (Fig 5A and 5B).

*S2/S1 ratio*: P1 gating was consistently affected by drug treatment [F(3,25) = 3.74, p = 0.027], whereas N1 ([F(3,25) = 3.23, p = 1.2] and P2 [F(3,25) = 1.5, p = 0.24] ratios were not altered. Post hoc tests indicated that amphetamine significantly disrupted P1 gating by increasing S2/S1 ratio due to a decrease in amplitude response to the first stimuli (Fig 5B). Pretreatment with PQ-10 failed to prevent disruption in gating (Fig 5C).

#### EEG oscillations

Amphetamine consistently enhanced aberrant EEG slow alpha oscillations in responses to both S1 and S2 auditory stimuli. Pretreatment with PQ-10 had no effect on those abnormalities (Fig 5D).

### 6. Effects of PQ-10 and PCP on AEP waveform components and oscillations

#### Evoked potentials and peak amplitudes

**Fig 6:**
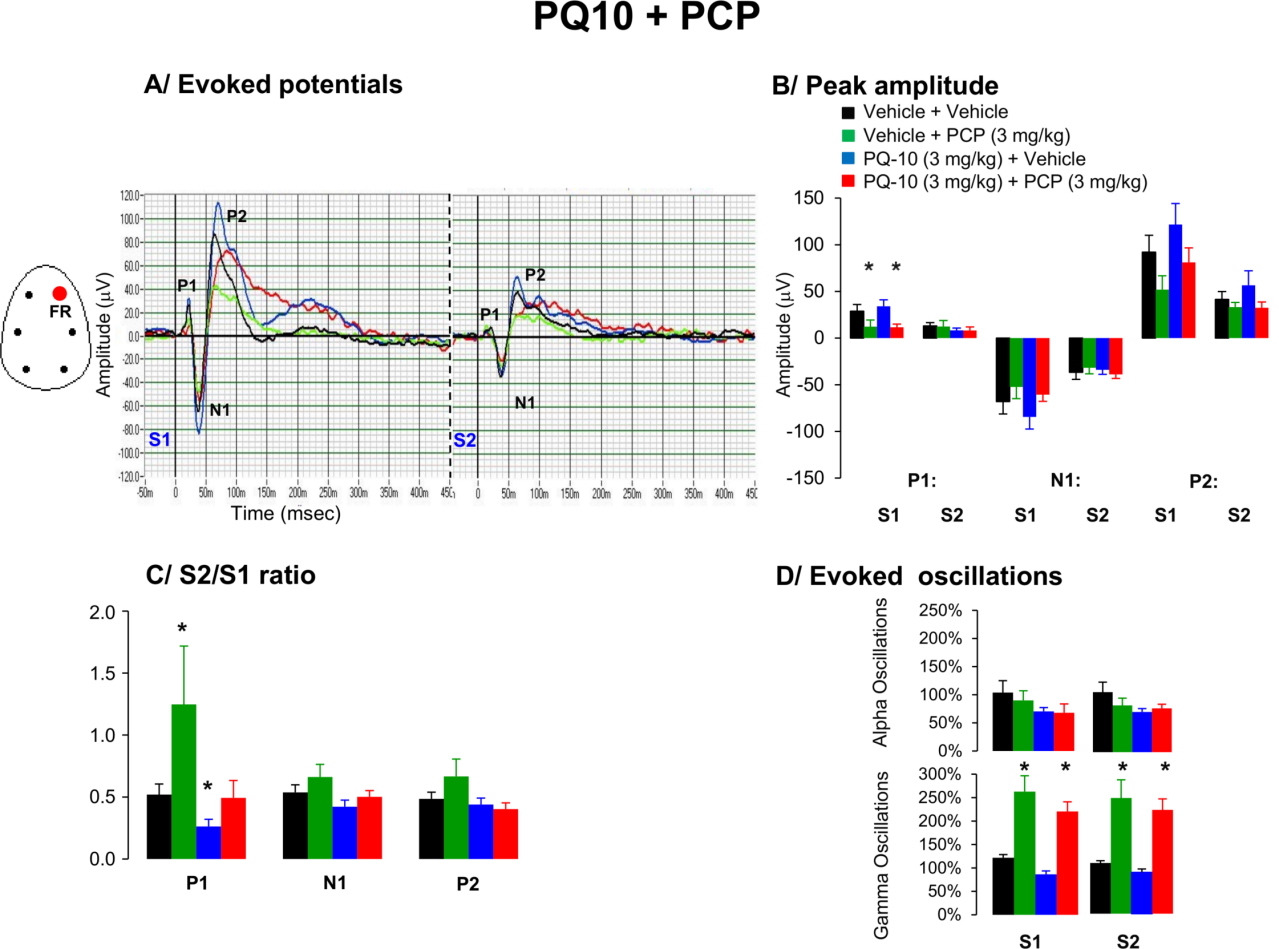
Effects of acute administration of PQ-10 (10 mg/kg), PCP (3 mg/kg) or in combination on A/ Grand average evoked potentials derived from frontal right hemisphere, B/ peak amplitude of the P1, N1, and P2 components (expressed in μV), C/ Mean S2/S1 ratio represent the gating index, D/ Bar graphs indicate average time frequency response in alpha and gamma EEG frequency oscillations from the right frontal cortex in awake motionless state for the period of 50–60 min following the second pharmacological treatment. Data are presented as mean ± S.E.M. of (n) animals for each condition (vehicle (6), PQ-10 (7), PCP (7) and PQ-10 + PCP (8)). 4 animals across different conditions with baseline S2/S1 ratio higher than 5 were discarded from the analysis. Administration of risperidone failed to improved PCP-induced disruption in gating response. * indicates significant difference from vehicle (p < 0.05).

There was a main drug effect on the peak amplitude to first stimuli for P1 [F (3,23) = 6.7, p = 0.0023], whereas peak amplitudes for N1 [F(3,23) = 2.7, p = 0.10] and P2 [F(3,23) = 3.42, p = 0.08] were slightly affected. Post hoc tests indicate that PCP significantly reduced P1 peak amplitude, whereas there was no consistent effect of PQ-10 on PCP-evoked reduction in P1 response (Fig 6A and 6B).

#### S2/S1 ratio

Evaluation of the S2/S1 ratio for the P1 component indicated that PCP consistently impaired this measure [F(3,23) = 10.1, p = 0.001]. PQ-10 consistently decreased S2/S1 ratio for P1, however the compound was without effect on PCP-induced deficits in this measure (Fig 6C).

#### EEG oscillations

PCP consistently enhanced aberrant high gamma oscillations in responses to both auditory S1 and S2 stimuli. Pretreatment with PQ-10 did not attenuate those abnormalities (Fig 6D).

### 7. Effects of LY404039 and amphetamine on AEP waveform components and oscillations

#### Evoked potentials and peak amplitudes

**Fig 7:**
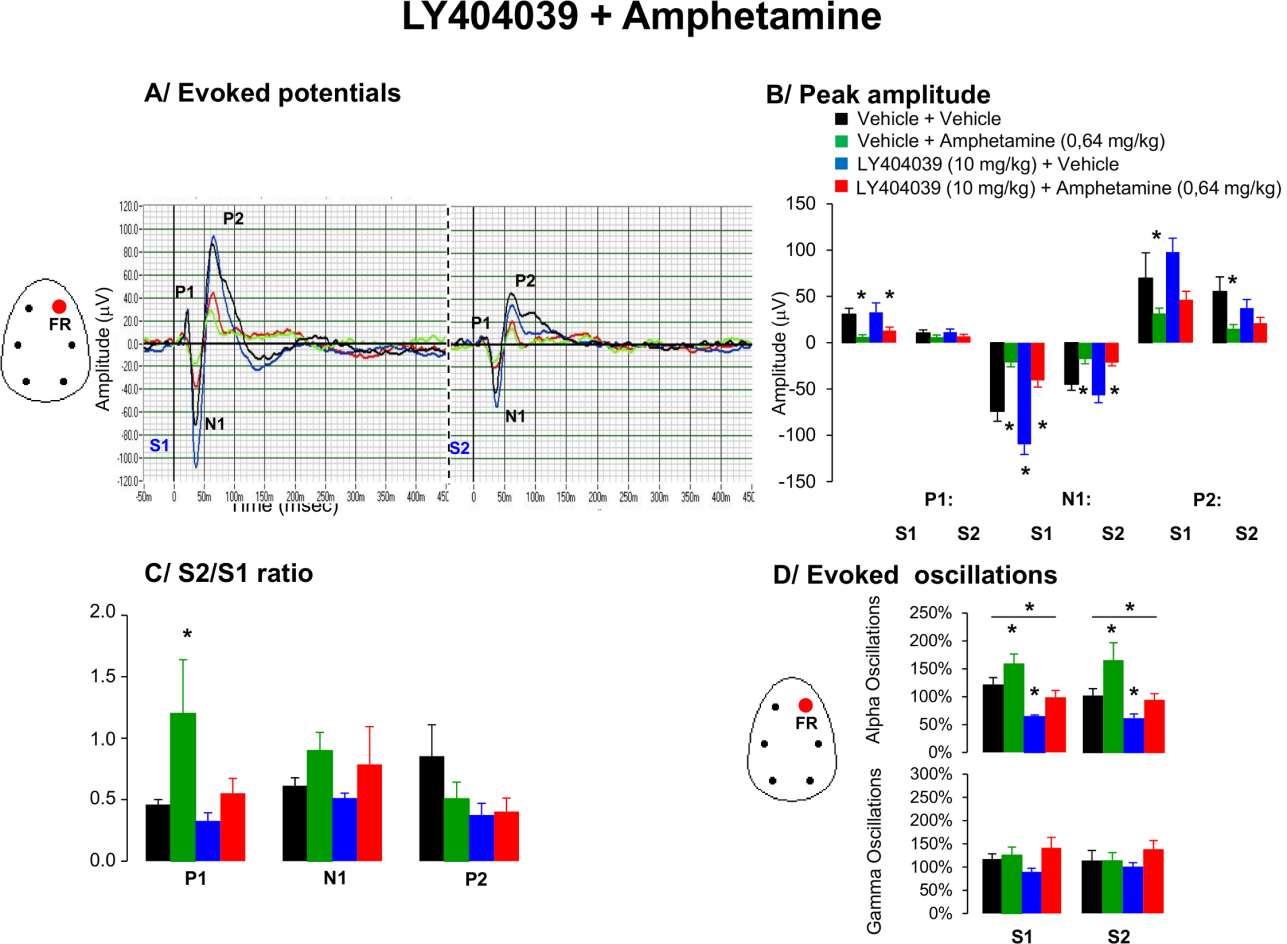
Effects of acute administration of LY404039 (10 mg/kg), amphetamine (0.64 mg/kg) or in combination on A/ Grand average evoked potentials derived from frontal right hemisphere, B/ peak amplitude of the P1, N1, and P2 components (expressed in μV), C/ Mean S2/S1 ratio represent the gating index, D/ Bar graphs indicate average time frequency response in alpha and gamma EEG frequency oscillations from the right frontal cortical in awake motionless periods for the time-interval of 50–60 min following the second pharmacological treatment. Data are presented as mean ± S.E.M. of (n) animals for each condition (vehicle (7), LY404039 (8), amphetamine (7) and LY404039 + amphetamine (8)). 2 animals across different conditions with baseline S2/S1 ratio higher than 5 were discarded from the analysis. Administration of LY404039 improved amphetamine-induced disruption in gating response. * indicates significant difference from vehicle (p < 0.05).

The grand average waveforms showed that P1, N1 and P2 amplitude responses to first stimuli were consistently affected by drug treatment [F(3,26) = 4.7, p = 0.01], [F(3,26) = 20.1, p = 0.00001] and [F(3,26) = 6.7, p = 0.009], respectively. Post hoc analysis revealed that amphetamine significantly decreased P1, N1 and P2 peak amplitudes, whereas LY404039 enhanced the amplitude of N1 (Fig 7A and 7B).

#### S2/S1 ratio

There was a main effect of drug on the S2/S1 ratio for P1 in the amphetamine treated group [F(3,26) = 3.1, p = 0.04], whereas N1 [F(3,26) = 1.1, p = 0.34] and P2 [F(3,26) = 2.0, p = 0.12] ratios were not affected. Post hoc analysis showed that amphetamine treatment significantly increased the S2/S1ratio for P1, which was reversed by LY404039 (Fig 7C).

*EEG oscillations*: Amphetamine enhanced aberrant slow alpha oscillations in responses to S1 and S2 auditory stimuli. Pretreatment with LY404039 attenuated those abnormalities (Fig 7D).

### 8. Effects of LY404039 and PCP on AEP waveform components and oscillations

#### Evoked potentials and peak amplitudes

**Fig 8:**
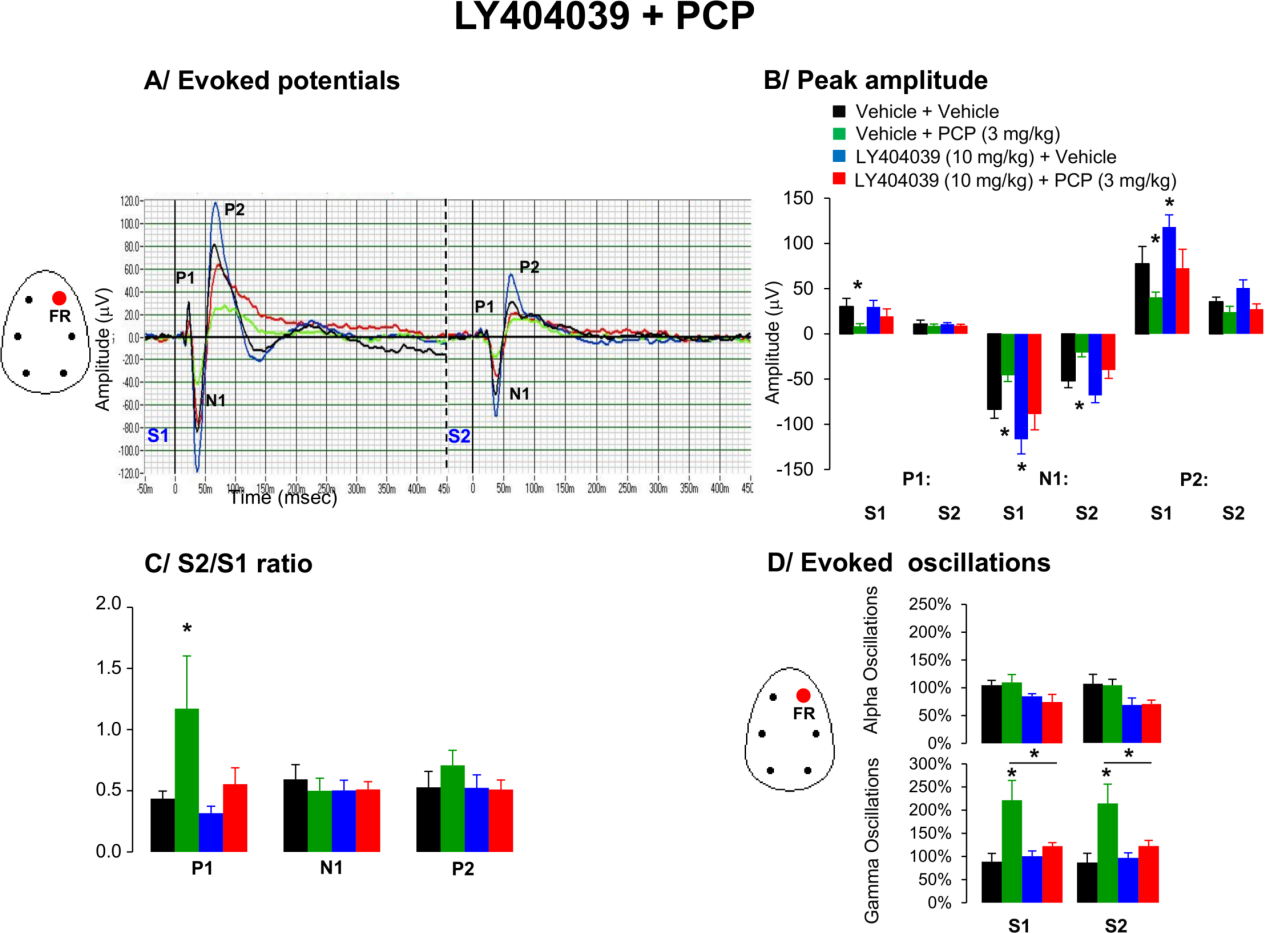
Effects of acute administration of LY404039 (10 mg/kg), PCP (3 mg/kg) or in combination on A/ Grand average evoked potentials derived from frontal right hemisphere, B/ peak amplitude of the P1, N1, and P2 components (expressed in μV), C/ Mean S2/S1 ratio represent the gating index, D/ Bar graphs showing EEG oscillations in alpha and gamma EEG frequency oscillations from the right frontal cortex in awake motionless periods for the time-interval of 50–60 min following the second pharmacological treatment. Data are presented as mean ± S.E.M. of (n) animals for each condition (vehicle (8), LY404039 (8), PCP (8) and LY404039 + PCP (8)). Administration of LY404039 improved PCP-induced disruption in gating response. * indicates significant difference from vehicle (p < 0.05).

The grand average waveforms shown in Fig 8A and peak measurements obtained in this paradigm indicated that P1 [F(3,36) = 3.4, p = 0.02], N1 [F(3,36) = 5.5, p = 0.003] and P2 [F(3,36) = 3.9, p = 0.02] amplitudes were affected by drug treatment. Post hoc analysis revealed that PCP consistently reduced both P1, N1 and P2 peak amplitudes (Fig 8B). In LY404039 treated animals, N1 and P2 peaks to first stimuli were significantly higher than the corresponding peaks to first stimuli in the vehicle treated group (Fig 8B).

#### S2/S1 ratio

There was a main drug effect on S2/S1 index for P1 [F(3,36) = 6.2, p = 0.002] (Fig 8C). Post hoc analysis indicated that PCP disrupted the S2/S1 for P1, an effect which was attenuated by LY404039 (Fig 8C).

#### EEG oscillations

Consistent alterations in higher gamma oscillations were observed with PCP in responses to both auditory S1 and S2 stimuli. Pretreatment with LY404039 attenuated PCP-induced aberrant gamma network oscillations in responses to both stimuli (Fig 8D).

## Discussion

The present study used auditory evoked potentials (AEP) and oscillations in conscious rats to confirm the efficacy of atypical antipsychotics in attenuating pharmacologically-induced deficits in sensory encoding and processing of auditory stimuli, and to address the potential of mGluR2 activation and of PDE10 inhibition as therapeutic strategies in conditions of auditory processing impairments. In the paired click paradigm, acute administration of PCP and amphetamine decreased the amplitude of S1 response and induced aberrant oscillations, which is associated with impaired initial encoding processes of auditory stimuli. Both risperidone and olanzapine reliably reduced PCP and amphetamine-induced alterations in peak amplitudes of AEP components, respectively. Activation of mGluR2 attenuated alterations in peak AEP amplitudes induced either by amphetamine or PCP, whereas PDE10 inhibition had no such effect.

### Altered dopamine and glutamate circuits affect AEP

In the present study, the AEP components measured under baseline conditions reflects sequential components in early auditory information processing, associated with suppression of S2 relative to S1 for P1, N1 and P2 components (Fig 1). Although the direct analogy between human and rat AEP components is unclear, this study and others support the rodent P50 paired click paradigm as a plausible analogue of human P50 suppression for studying the integrity of brain inhibitory circuits and evaluation of treatments [53–57].

Hyperdopaminergic and hypoglutamatergic transmission has been linked to cognitive deficits in schizophrenia [58,59]. Amphetamine and the NMDA receptor antagonists PCP, ketamine and MK801 have repeatedly been used to model schizophrenia-like deficits in sensory encoding and information processing in both humans and animals. In the present work, both amphetamine and PCP disrupted the S2/S1 ratio for P1 and N1 peaks due to reduced suppression of the response to the first stimuli, which is in line with previous reports showing a consistent reduction in both the amplitude to the first stimuli and the gating index [60–62]. Deficits in P50 suppression by reduced S1 amplitude has been reported in unmedicated schizophrenic patients [41,14,63]. The present data may suggest that PCP and amphetamine-induced deficits in the ability to ‘gate in’ information and in the perception threshold of auditory stimuli may resemble the findings in unmedicated schizophrenic patients.

### Atypical antipsychotics attenuate disruption in AEP and aberrant EEG oscillations

Clinical studies have demonstrated efficacy of the second-generation antipsychotics to reverse deficits in gating [4,20]. Olanzapine, which exhibits a potent antagonist activity at the 5-HT2 receptor, elevated the amplitude response S1 stimuli suggesting an opposite effect might restore deficits of P50 suppression in rodents [54]. In accordance with this model, the present work demonstrated that both atypical antipsychotics risperidone and olanzapine increased peak amplitudes to S1 stimuli and attenuated amphetamine and PCP-induced altaerations in AEP peak amplitudes, respectively. The results suggest potential efficacy to improve pharmacologically-induced impairments in auditory processing.

In the present study, a transient pathological enhancement in gamma network oscillations was elicited by PCP. Our results extend previous observations on enhanced aberrant gamma oscillations following blockade of NMDA receptors and suggest that disruption in encoding processes of auditory stimuli may be confounded by the abnormal enhancement of gamma activity.

Hallucinogenic drugs such as lysergic acid diethylamide (LSD) and the serotonergic phenethylamine hallucinogen [–]-2,5-dimethoxy-4-methylamphetamine (DOM) increased the power in the alpha frequency oscillations [64]. The atypical antipsychotics decreased high frequency as well as slow alpha oscillations in humans and animals [65–68]. Amphetamine is widely used to recreate positive symptoms of schizophrenia in rodents, and this drug increases motor activity, during which the firing rate of neurons in the hippocampus rhythmically increased and decreased at ~8 Hz “theta rhythm” [69]. In the present work, amphetamine enhanced a transient aberrant EEG slow alpha oscillations during motionless behavior in responses to both S1 and S2 stimuli. Accordingly, it has been suggested that alpha rhythm may emerge during loss of attention in immobile state or during failure to process sensory input, and may serve to functionally disengage and reduce the processing capabilities of a given brain region [70]. Therefore, it is hypothesized that the alpha activity elicicted by amphetamine may silence other frequency rhythms to disengage and/or inhibit network computations of those cortical regions during encoding processes.

The disruption in dopaminergic and/or glutamatergic transmission may results in abnormal networks oscillations observed in schizophrenics, and antipsychotics-induced attenuation of aberrant alpha and gamma network oscillations can provide significant benefits for information processing. In line with these observations, pretreatment with the atypical antipsychotics risperidone and olanzapine consistently reduced PCP and amphetamine-induced transient pathological enhanced higher gamma and alpha frequency oscillations.

### PQ-10 failed to prevent disruptive effects on AEP and aberrant EEG oscillations

In schizophrenia, increases in dopamine D2 receptors and decreased D1 receptor activities may lead to reduced cAMP levels in frontal and temporal cortex, leading to psychosis and cognitive deficits. Recently, it has been hypothesized that inhibition of dopaminergic signaling through PDE10A could represent a novel non-receptor-based mechanism for the reduction of psychosis and related cognitive dysfunction in schizophrenia [40,41,71].

Here, PQ-10 at the dose of 3 mg/kg reduced the S2/S1 ratio for the P1 amplitude in the initial dose response and combined pharmacological studies: this dose is highly brain penetrant with a brain-plasma ratio of 0.66 at 30 min after oral administration and well above the behaviorally active dose of 1 mg/kg in the MK801 model [72]. At this dose PQ-10 enhanced the amplitude of P1, N1 and P2 components of AEPs to the first stimuli (as already suggested above), however, it did not attenuate the detrimental effects of amphetamine and PCP on evoked potentials and S2/S1 ratios for P1. The present data are in agreement with a recent AEP study showing that PQ-10 enhanced the N1 amplitude to the first stimulus in the cortex of rats [73]. Of note, a consistent deficit effects of PCP on S2/S1 ratio for P1 was found in all experiments, however the effect of PCP on S2/S1 ratio for P2 was not consistently altered. It is likely that the response variability to S1 for P2 amplitude caused this discrepancy between studies; however it cannot be ruled out that PQ-10 could attenuate deficit in the S2/S1 amplitude as it was repeatedly found that PQ-10 increase peak amplitude to S1 for N1 and P2 components, which represent later stages of information processing. In contrast, intravenous administration of another selective PDE10 inhibitor TP-10 reversed amphetamine-induced gating impairments in anesthetized rats [74], suggesting that the route of administration as well as the model used (freely moving vs. anesthetized rats), might cause discrepancies between studies.

The inhibition of PDE10A showed mixed results on cognition. In the scopolamine and MK801 models, PQ-10 showed efficacy to reverse deficits in the object recognition memory at the doses of 0.3 and 1 mg/kg, respectively [72], while the selective PDE10A inhibitor MP-10 reversed an MK-801-induced memory deficit in social odor recognition in mice [70]. However, PDE10A deficient mice showed no alteration in learning and memory performance in the passive avoidance and water escape tasks [24,73]. In contrast to amphetamine and PCP-induced locomotor activity, PDE10 inhibitors have been shown to reduce locomotor activity and had no effect on exploratory behavior [71,74]. Thus, it is likely that the lack of efficacy of PQ-10 on amphetamine and PCP-induced gating deficits could partially be explained by altered motor behavior: behavioral cognitive measures could be more sensitive to assess PDE10A inhibition as compared to neurophysiological read-outs.

It is also important to note that another phosphodiesterase inhibitor, PDE4 rolipram, reversed amphetamine-induced abnormalities in auditory evoked potentials in mice [75] suggesting that enhancement of evoked potentials might be a general feature of increased intracellular cAMP/cGMP second messengers through PDE inhibition. Overall, PQ-10 failed to restore amphetamine and PCP-induced gating deficits in conscious rats, and suggests that impairments in sensory processing are not sensitive to changes in the levels of intracellular cAMP/cGMP elicited in the amphetamine and PCP psychosis models.

### LY404039 normalized disruption of AEP and aberrant cortical gamma oscillations

mGluR2 orthosteric agonists potently attenuate both amphetamine and PCP-evoked hyperactivity, show antipsychotic-like effects in a variety of preclinical rodent paradigms, and suppress ketamine-induced aberrant augmentation in gamma oscillations [7,48,66,76,77]. In addition, the prodrug LY2140023 significantly improves both positive and negative symptoms of schizophrenia [50], which provides further evidence that modulation of glutamate tone may have antipsychotic properties to treat refractory clinical symptoms of schizophrenia.

The present studies revealed consistent effects of the mGluR2 agonist in two different animal models predictive of antipsychotic potential. Amphetamine and PCP yielded significant reduction of evoked potentials, whereas LY404039 at the dose that reduced the S2/S1 ratio for the P1 amplitude enhanced the auditory response to the first stimuli, indicating that the mGluR2 orthosteric agonist could increase the initial encoding response of an auditory stimulus. Moreover, LY404039 restored the gating deficits induced by amphetamine and PCP, an effect shared by atypical antipsychotics.

A functional interaction between mGluR2 and 5-HT2A receptors has been demonstrated to form a heterodimer complex in the prefrontal cortex [78]. PCP-induced glutamate release through 5-HT and 5-HT2A receptor mechanisms might result in dishinbition of pyramidal neurons, leading to abnormal network gamma oscillations. Given that atypical antipsychotics display a robust antagonism activity at 5-HT2A receptors, we hypothesize that activation of mGluR2 may exert a negative control and act to reduce glutamate release following the activation of 5-H2A receptors in prefrontal cortical areas, then leading to improved information processing. In the present work, LY404039 significantly attenuated amphetamine and PCP-induced disruption in frontal network gamma oscillations, which is in line with recent reports showing efficacy of mGluR2 drugs to inhibit MK-801 and ketamine-induced excessive gamma oscillations [66,76]. MGluR2 signaling may alleviate auditory processing deficiencies and attenuate abnormalities in large EEG networks partially through different mechanisms from those of atypical antipsychotics. However, the recent binding study revealed that LY404039 displayed relatively high affinity towards dompaminergic D2 receptors as this compound inhibited the binding of the specific dopamine D2 receptor antagonist, [^3^H]domperidone to human cloned D2 receptors [79]. Therefore, it should be considered that clinical antipsychotic action of LY404039 may depend on synergistic stimulation of glutamate receptors with a partial agonism at the dopamine D2 receptors, thus reducing endogenous dopaminergic neurotransmission.

### P50 gating versus pre-pulse inhibition (PPI)

Two paradigms are widely used to investigate sensory gating response: the behavioral prepulse inhibition of the startle response (PPI) and the neurophysiological P50 gating. PPI and P50 measures may have overlapping as well as separate neurobiological substrates.

PPI is a motor startle reflex elicited by a sudden and strong sensory stimulus, usually auditory. This reflex can be reduced by a weaker stimulus that precedes the startle-eliciting stimulus [80]. Modulation of the startle reflex (including phenomena such as habituation, sensitization and fear potentiation) involves a number of brain structures located up to the forebrain, i.e. auditory nerve, ventral cochlear nucleus, dorsal nucleus of the lateral lemniscus, caudal pontine reticular nucleus, spinal motor neurons [81–83].

In a “paired-stimulus” paradigm two identical auditory stimuli are presented 500 ms apart to evaluate neurophysiological sensory gating, reflecting an individual’s ability to screen out trivial or repetitive stimuli in order to protect against information overload. The brain neuronal structures associated with the dynamic generation and suppression of P50 gating have been identified by advanced brain mapping with neurophysiological methods that facilitate detection of activity from deeper brain sources (e.g. fMRI or magnetoencephalography) [84]. The superior temporal gyrus, hippocampus, dorsolateral prefrontal cortex, and thalamus are neural structures that contribute to the generation of a P50 response. Unlike the PPI startle reflex, P50 suppression has a substrate that is easily identified across animal species, such as N34.

Some reports indicate that PDE10A inhibitors reverse pharmacologically -induced (e.g. MK-801, quinpirole; apomorphine/SCH23390) deficits in PPI [85,86]. These findings contrast with the findings of PQ-10 in the current study. Discrepancies were also reported regarding the effect of mGluR2 activation in amphetamine and PCP-induced PPI deficit paradigm [87]. In the present study, the potential of mGluR2 but not PDE10 to improve P50 gating disruption may reflect distinct parametric sensitivities and/or distinct neural mechanisms of inhibition. Consistent with earlier studies on the relationship between PPI and P50 suppression in either the auditory or visual modality [88,89], no correlations could be derived between these two gating measures in earlier PPI and the present P50 gating paradigms. These observations further emphasize that the sensory gating P50 and sensorimotor gating PPI measures reflect different underlying phenomena [90].

### AEP paradigm: application and translation suitability

Auditory potentials have mostly been studied in anesthetized animals and in different inbred mice [91,92]. In contrast, the current study used pharmacological challenges in conscious rats to closely model gating deficits and aberrant network oscillations as found in schizophrenics. This approach offers valuable opportunities to probe the integrity of sensory processing networks and to study the mechanisms of potential drug actions in translational research.

Collectively, our results demonstrate that modulation of mGluR2 signaling can effectively alleviate deficits in pre-attentive stages of sensory information processing in two rat models of schizophrenia, whereas PDE10 inhibition had no such effect. The results provide a basis for further validation of this hypothesis in other animal models, and ultimately in clinical trials.

## References

[1] Butler PD, Javitt DC. Early-stage visual processing deficits in schizophrenia. Curr Opin Psychiatry 2005; 2:151–157. 10.1097/00001504-200503000-00008 PMC1994776 16639168

[2] Javitt DC, Shelley AM, Silipo G, Lieberman JA. Deficits in auditory and visual context-dependent processing in schizophrenia: defining the pattern. Arch Gen Psychiatry 2000; 12:1131–1137. 10.1001/archpsyc.57.12.1131 11115326

[3] Adler LE, Pachtman E, Franks RD, Pecevich M, Waldo MC, Freedman R. Neurophysiological evidence for a defect in neuronal mechanisms involved in sensory gating in schizophrenia. Biol Psychiatry 1982; 6:639–654. 7104417

[4] Light GA, Braff DL. Do self-reports of perceptual anomalies reflect gating deficits in schizophrenia patients? Biol Psychiatry 2000; 47:463–467. 10704957 10.1016/s0006-3223(99)00280-2

[5] Joy B, McMahon RP, Shepard PD. Effects of acute and chronic clozapine on D-amphetamine-induced disruption of auditory gating in the rat. Psychopharmacology 2004; 2:274–282. 10.1007/s00213-003-1731-4 14726994

[6] Cromwell HC, Mears RP, Wan L, Boutros NN. Sensory gating: a translational effort from basic to clinical science. Clin EEG Neurosci. 2008; 39:69–72. 18450171 10.1177/155005940803900209 PMC4127047

[7] Schoepp DD, Jane DE, Monn JA. Pharmacological agents acting at subtypes of metabotropic glutamate receptors. Neuropharmacology 1999; 38:1431–1476. 10530808 10.1016/s0028-3908(99)00092-1

[8] Boutros NN, Brockhaus-Dumke A, Gjini K, Vedeniapin A, Elfakhani M, Burroughs S et al Sensory-gating deficit of the N100 mid-latency auditory evoked potential in medicated schizophrenia patients. Schizophr Res. 2009; 113: 339–46. 10.1016/j.schres.2009.05.019 19524407 PMC2734408

[9] Cullum CM, Harris JG, Waldo MC, Smernoff E, Madison A, Nagamoto HT et al Neurophysiological and neuropsychological evidence for attentional dysfunction in schizophrenia. Schizophr Res. 1993: 10: 131–141. 8398945 10.1016/0920-9964(93)90048-n

[10] Erwin RJ, Turetsky BI, Moberg P, Gur RC, Gur RE. P50 abnormalities in schizophrenia: relationship to clinical and neuropsychological indices of attention. Schizophr Res. 1998; 33: 157–67. 9789908 10.1016/s0920-9964(98)00075-9

[11] Lijffijt M, Lane SD, Meier SL, Boutros NN, Burroughs S, Steinberg JL et al P50, N100, and P200 sensory gating: relationships with behavioral inhibition, attention, and working memory. Psychophysiology. 2009; 46:1059–68. 10.1111/j.1469-8986.2009.00845.x 19515106 PMC2821570

[12] Mazhari S, Price G, Waters F, Dragović M, Jablensky A.Evidence of abnormalities in mid-latency auditory evoked responses (MLAER) in cognitive subtypes of patients with schizophrenia. Psychiatry Res. 2011; 187:317–23. 10.1016/j.psychres.2011.01.003 21292328

[13] Clementz BA, Geyer MA, Braff DL. P50 suppression among schizophrenia and normal comparison subjects: a methodological analysis. Biol Psychiatry. 1997; 41:1035–44. 9129784 10.1016/S0006-3223(96)00208-9

[14] Freedman R, Adler LE, Waldo MC, Pachtman E, Franks RD. Neurophysiological evidence for a defect in inhibitory pathways in schizophrenia: comparison of medicated and drug-free patients. Biol Psychiatry 1983; 18: 537–551. 6134559

[15] Blumenfeld LD, Clementz BA. Response to the first stimulus determines reduced auditory evoked response suppression in schizophrenia: single trials analysis using MEG. Clin Neurophysiol. 2001;112:1650–9. 11514248 10.1016/s1388-2457(01)00604-6

[16] Clementz BA, Dzau JR, Blumenfeld LD, Matthews S, Kissler J. Ear of stimulation determines schizophrenia-normal brain activity differences in an auditory paired-stimuli paradigm. Eur J Neurosci. 2003; 18:2853–8. 14656334 10.1111/j.1460-9568.2003.03027.x

[17] Johannesen JK, Kieffaber PD, O'Donnell BF, Shekhar A, Evans JD, Hetrick WP. Contributions of subtype and spectral frequency analyses to the study of P50 ERP amplitude and suppression in schizophrenia. Schizophr Res. 2005; 78: 269–84 16002265 10.1016/j.schres.2005.05.022

[18] Boutros NN, Belger A. Midlatency evoked potentials attenuation and augmentation reflect different aspects of sensory gating. Biol Psychiatry. 1999; 45: 917–22. 10202580 10.1016/s0006-3223(98)00253-4

[19] Adler LE, Rose G, Freedman R. Neurophysiological studies of sensory gating in rats: effects of amphetamine, phencyclidine, and haloperidol. Biol Psychiatry 1986; 21:787–98 3730461 10.1016/0006-3223(86)90244-1

[20] Adler LE, Olincy A, Cawthra EM, McRae KA, Harris JG, Nagamoto HT et al Varied effects of atypical neuroleptics on P50 auditory gating in schizophrenia patients. Am J Psychiatry 2004; 161:1822–1828. 15465979 10.1176/ajp.161.10.1822

[21] Cadenhead KS, Light GA, Geyer MA, Braff DL. Sensory gating deficits assessed by the P50 event-related potential in subjects with schizotypal personality disorder. Am J Psychiatry 2000;157:55–9. 10618013 10.1176/ajp.157.1.55

[22] Ellenbroek BA. Pre-attentive processing and schizophrenia: animal studies. Psychopharmacology 2004; 174:65–74. 15205880 10.1007/s00213-003-1684-7

[23] Gruber T, Müller MM. Oscillatory brain activity dissociates between associative stimulus content in a repetition priming task in the human EEG. Cereb Cortex 2005; 1:109–116. 10.1093/cercor/bhh113 15238442

[24] Tallon-Baudry C. Attention and awareness in synchrony. Trends Cogn Sci. 2004; 12: 523–525. 10.1016/j.tics.2004.10.008 15556020

[25] Herrmann CS, Demiralp T. Human EEG gamma oscillations in neuropsychiatric disorders. Clinical Neurophysiology 2005; 116:2719–2733. 16253555 10.1016/j.clinph.2005.07.007

[26] Gandal MJ, Edgar JC, Klook K, Siegel SJ. Gamma synchrony: Towards a translational biomarker for the treatment-resistant symptoms of schizophrenia. Neuropharmacology 2011; 62:1504–18. 10.1016/j.neuropharm.2011.02.007 21349276 PMC3264822

[27] Lee KH, Breakspear WLM, Gordon ME. Synchronous gamma activity: A review and contribution to an integrative neuroscience model of schizophrenia. Brain Res Rev. 2003; 41:57–78. 12505648 10.1016/s0165-0173(02)00220-5

[28] Phillips WA, Silverstein SM. Convergence of biological and psychological perspectives on cognitive coordination in schizophrenia. Behav Brain Sci. 2003; 26:65–82 14598440 10.1017/s0140525x03000025

[29] Spencer KM, Nestor PG, Perlmutter R, Niznikiewicz MA, Klump MC, Frumin M et al Neural synchrony indexes disordered perception and cognition in schizophrenia. Proc Natl Acad Sci USA. 2004; 101:17288–17293. 15546988 10.1073/pnas.0406074101 PMC535363

[30] Uhlhaas PJ, Singer W. Neural synchrony in brain disorders: relevance for cognitive dysfunctions and pathophysiology. Neuron 2006; 52:155–168. 17015233 10.1016/j.neuron.2006.09.020

[31] Uhlhaas PJ, Singer W. Abnormal neural oscillations and synchrony in schizophrenia. Nat Rev Neurosci 2010; 11:100–113 10.1038/nrn2774 20087360

[32] Baldeweg T, Spence S, Hirsch SR, Gruzelier J. Gamma-band electroencephalographic oscillations in a patient with somatic hallucinations. Lancet 1998; 352:620–621. 10.1016/S0140-6736(05)79575-1 9746027

[33] Spencer KM. Baseline gamma power during auditory steady-state stimulation in schizophrenia. Front Hum Neurosci. 2011; 5: 190 10.3389/fnhum.2011.00190 22319485 PMC3267371

[34] Spencer KM, Salisbury DF, Shenton ME, McCarley RW. Gamma-band auditory steady-state responses are impaired in first episode psychosis. Biol Psychiatry 2008; 64: 369–375. 10.1016/j.biopsych.2008.02.021 18400208 PMC2579257

[35] Pinault D. N-methyl d-aspartate receptor antagonists ketamine and MK-801 induce wake-related aberrant gamma oscillations in the rat neocortex. Biol Psychiatry 2008; 63:730–735. 18022604 10.1016/j.biopsych.2007.10.006

[36] Ma J, Leung LS. The supramammillo-septal-hippocampal pathway mediates sensorimotor gating impairment and hyperlocomotion induced by MK-801 and ketamine in rats. Psychopharmacology 2007; 191: 961–974. 17219218 10.1007/s00213-006-0667-x

[37] Kittelberger K, Hur EE, Sazegar S, Keshavan V, Kocsis B. Comparison of the effects of acute and chronic administration of ketamine on hippocampal oscillations: relevance for the NMDA receptor hypofunction model of schizophrenia. Brain Struct Funct. 2011; 217:395–409. 10.1007/s00429-011-0351-8 21979451 PMC3288729

[38] Kocsis B. Differential role of NR2A and NR2B subunits in N-methyl-D-aspartate receptor antagonist-induced aberrant cortical gamma oscillations. Biol Psychiatry 2012; 71:987–995. 10.1016/j.biopsych.2011.10.002 22055014 PMC3276718

[39] Laruelle M, Kegeles LS, Abi-Dargham A. Glutamate, dopamine, and schizophrenia: from pathophysiology to treatment. Ann N Y Acad Sci. 2003; 1003:138–58. 14684442 10.1196/annals.1300.063

[40] Menniti FS, Chappie TA, Humphrey JM, Schmidt CJ. Phosphodiesterase 10A inhibitors: a novel approach to the treatment of the symptoms of schizophrenia. Curr Opin Investig Drugs 2007; 8: 54–59. 17263185

[41] Seeger TF, Bartlett B, Coskran TM, Culp JS, James LC, Krull DL et al Immunohistochemical localization of PDE10A in the rat brain. Brain Res. 2003; 985:113–126. 12967715 10.1016/s0006-8993(03)02754-9

[42] Xie Z, Adamowicz WO, Eldred WD, Jakowski AB, Kleiman RJ, Morton DG et al Cellular and subcellular localization of PDE10A, a striatum-enriched phosphodiesterase. Neurosciences 2006; 139:597–607. 10.1016/j.neuroscience.2005.12.042 PMC1464838 16483723

[43] Siuciak JA, Chapin DS, Harms JF, Lebel LA, McCarthy SA, Chambers L et al Inhibition of the striatum-enriched phosphodiesterase PDE10A: a novel approach to the treatment of psychosis. Neuropharmacology 2006; 51:386–396. 16780899 10.1016/j.neuropharm.2006.04.013

[44] Coyle JT. Glutamate and schizophrenia: beyond the dopamine hypothesis. Cell Mol Neurobiol. 2006; 4:365–84. 10.1007/s10571-006-9062-8 PMC11881825 16773445

[45] Javitt DC. Glutamate and schizophrenia: phencyclidine, N-methyl-D-aspartate receptors, and dopamine-glutamate interactions. Int Rev Neurobiol. 2007; 78:69–108. 17349858 10.1016/S0074-7742(06)78003-5

[46] Lahti AC, Weiler MA, Tamara-Michaelidis BA, Parwani A, Tamminga CA. Effects of ketamine in normal and schizophrenic volunteers. Neuropsychopharmacology 2001; 4:455–467. 10.1016/S0893-133X(01)00243-3 11557159

[47] Geyer MA, Krebs-Thomson K, Braff DL, Swerdlow NR. Pharmacological studies of prepulse inhibition models of sensorimotor gating deficits in schizophrenia: a decade in review. Psychopharmacology 2001; 2:117–54. 10.1007/s002130100811 11549216

[48] Rorick-Kehn LM, Johnson BG, Burkey JL, Wright RA, Calligaro DO, Marek GJ et al Pharmacological and pharmacokinetic properties of a structurally novel, potent, and selective metabotropic glutamate 2/3 receptor agonist: in vitro characterization of agonist (-)-(1R,4S,5S,6S)-4-amino-2-sulfonylbicyclo[3.1.0]-hexane-4,6-dicarboxylic acid (LY404039). J Pharmacol Exp Ther. 2007; 321:308–17. 17204749 10.1124/jpet.106.110809

[49] Cartmell J, Monn JA, Schoepp DD. Attenuation of specific PCP-evoked behaviors by the potent mGlu2/3 receptor agonist, LY379268 and comparison with the atypical antipsychotic, clozapine. Psychopharmacology. 2000; 148:423–9. 10928316 10.1007/s002130050072

[50] Patil ST, Zhang L, Martenyi F, Lowe SL, Jackson KA, Andreev BV et al Activation of mGlu2/3 receptors as a new approach to treat schizophrenia: a randomized Phase 2 clinical trial Nat Med. 2007; 9:1102–1107. 10.1038/nm1632 17767166

[51] Siuciak JA, McCarthy SA, Chapin DS, Fujiwara RA, James LC, Williams RD et al Genetic deletion of the striatum-enriched phosphodiesterase PDE10A: evidence for altered striatal function. Neuropharmacology 2006; 51:374–85. 16769090 10.1016/j.neuropharm.2006.01.012

[52] Paxinos G, Watson C. The Rat Brain in Stereotaxic Coordinates Academic Press, San Diego, USA 1998.

[53] Connolly PM, Maxwell CR, Kanes SJ, Abel T, Liang Y, Tokarczyk J et al Inhibition of auditory evoked potentials and prepulse inhibition of startle in DBA/2J and DBA/2Hsd inbred mouse substrains. Brain Res. 2003; 992:85–95. 14604776 10.1016/j.brainres.2003.08.035

[54] Maxwell CR, Liang Y, Weightman BD, Kanes SJ, Abel T, Gur RE et al Effects of chronic olanzapine and haloperidol differ on the mouse N1 auditory evoked potential. Neuropsychopharmacology 2004; 29:739–46. 14735128 10.1038/sj.npp.1300376

[55] Phillips JM, Ehrlichman RS, Siegel SJ. Mecamylamine blocks nicotine-induced enhancement of the P20 auditory event-related potential and evoked gamma. Neuroscience 2007; 144: 1314–1323. 17184927 10.1016/j.neuroscience.2006.11.003 PMC1868669

[56] Siegel SJ, Maxwell CR, Majumdar S, Trief DF, Lerman C, Gur RE et al Monoamine reuptake inhibition and nicotine receptor antagonism reduce amplitude and gating of auditory evoked potentials. Neuroscience 2005; 133:729–738. 15908134 10.1016/j.neuroscience.2005.03.027

[57] Umbricht D, Vyssotky D, Latanoy A, Nitsch R, Brambilla R, D’Adamo P et al Midlatency auditory event-related potentials in mice: comparison to midlatency auditory ERPs in humans. Brain Res. 2004; 1019:189–200. 15306253 10.1016/j.brainres.2004.05.097

[58] Barch DM, Ceaser A. Cognition in schizophrenia: core psychological and neural mechanisms. Trends Cog Sci. 2012; 16:27–34. 10.1016/j.tics.2011.11.015 PMC3860986 22169777

[59] Lewis D, Moghaddam B. Cognitive dysfunction in schizophrenia: convergence of gamma-aminobutyric acid and glutamate alterations. Arch Neurol. 2006; 63:1372–1376. 17030651 10.1001/archneur.63.10.1372

[60] De Bruin NM, Ellenroek BA, Coenen AM, Van Luitelaar EL. Differential effects of ketamine on gating of auditory evoked potentials and prepulse inhibition in rats. Psychopharmacology 1999; 142: 9–17. 10102777 10.1007/s002130050856

[61] Johnson MR, Adler LE. Transient impairment in P50 auditory sensory gating induced by a cold-pressor test. Biol Psychiatry 1993; 33:380–387. 8386006 10.1016/0006-3223(93)90328-b

[62] Maxwell CR, Kanes SJ, Abel T, Siegel SJ. Phosphodiesterase inhibitors: a novel mechanism for receptor-independent antipsychotic medications. Neuroscience 2004; 129:101–107. 15489033 10.1016/j.neuroscience.2004.07.038

[63] Patterson JV, Jin Y, Gierczak M, Hetrick WP, Potkin S, Bunney WE et al Effects of temporal variability on P50 and the gating ratio in schizophrenia: a frequency domain adaptive filter single-trial analysis. Arch Gen Psychiatry 2000; 57:57–64 10632233 10.1001/archpsyc.57.1.57

[64] Dimpfel W, Spüler M, Nichols DE. Hallucinogenic and stimulatory amphetamine derivatives: fingerprinting DOM, DOI, DOB, MDMA, and MBDB by spectral analysis of brain field potentials in the freely moving rat (Tele-Stereo-EEG). Psychopharmacology 1989; 98:297–303. 2568652 10.1007/BF00451678

[65] Dimpfel W. Characterization of atypical antipsychotic drugs by a late decrease of striatal alpha1 spectral power in the electropharmacogram of freely moving rats. Br J Pharmacol. 2007; 152: 538–548. 17700713 10.1038/sj.bjp.0707427 PMC2050823

[66] Jones NC, Reddy M, Anderson P, Salzberg M, O’Brien T, Pinault D. Acute administration of typical and atypical antipsychotics reduces EEG gamma power, but only the preclinical compound LY379268 reduces the ketamine-induced rise in gamma power. Int J Neuropsychopharmacol. 2011; 15:657–668. 10.1017/S1461145711000848 21733235 PMC3353488

[67] Lacroix D, Chaput Y, Rodriguez JP, Filion M, Morrison D, St-Denis P et al Quantified EEG changes associated with a positive clinical response to clozapine in schizophrenia. Prog Neuropsychoparmacol Biol Psychiatry 1995; 19:961–876. 10.1016/0278-5846(95)00116-d 8539424

[68] Saletu B, Grunberger J, Linzmayer L, Anderer P. Comparative placebo-controlled pharmacodynamic studies with zotepine and clozapine utilizing pharmaco-EEG and psychometry. Pharmacopsychiatry 1987; 20:12–27. 2883677 10.1055/s-2007-1017125

[69] Vanderwolf CH. Hippocampal electrical activity and voluntary movement in the rat. Electroencephalogr Clin Neurophysiol. 1969; 26: 407–418. 4183562 10.1016/0013-4694(69)90092-3

[70] Klimesch W, Sauseng P, Hanslmavr S. EEG alpha oscillations: the inhibition-timing hypothesis. Brain Res Rev. 2007; 53: 63–88. 16887192 10.1016/j.brainresrev.2006.06.003

[71] Grauer SM, Pulito VL, Navarra RL. PDE10A inhibitor activity in preclinical models of the positive, cognitive and negative symptoms of schizophrenia. J Pharmacol Exp Ther. 2009; 331:574–90. 10.1124/jpet.109.155994 19661377

[72] Reneerkens OA, Rutten K, Bollen E, Hage T, Blokland A, Steinbusch HW et al Inhibition of phoshodiesterase type 2 or type 10 reverses object memory deficits induced by scopolamine or MK-801. Behav Brain Res. 2013a; 236:16–22. 22951181 10.1016/j.bbr.2012.08.019

[73] Reneerkens OA, Sambeth A, Blokland A, Prickaerts J. PDE2 and PDE10, but not PDE5, inhibition affect basic auditory information processing in rats. Behav Brain Res. 2013b; 250:251–256. 23688598 10.1016/j.bbr.2013.05.014

[74] Schmidt CJ, Chapin DS, Cianfrogna J, Corman ML, Hajos M, Harms JF et al Preclinical characterization of selective phosphodiesterase 10A inhibitors: a new therapeutic approach to the treatment of schizophrenia. J Pharmacol Exp Ther. 2008; 325:681–690. 10.1124/jpet.107.132910 18287214

[75] Halene TB, Siegel SJ. Antipsychotic-like properties of phosphodiesterase 4 inhibitors: Evaluation of 4-(3-Butoxy-4-methoxybenzyl)-2-imidazolidinone (RO-20-1724) with auditory event-related potentials and prepulse inhibition of startle. J Pharmacol Exp Ther. 2008; 326:230–239. 10.1124/jpet.108.138586 18420599

[76] Hiyoshi T, Marumo T, Hikichi H, Tomishima Y, Urabe H, Tamita T et al Neurophysiologic and antipsychotic profiles of TASP0433864, a novel positive allosteric modulator of metabotropic glutamate 2 receptor. J Pharmacol Exp Ther. 2014; 351:642–53. 10.1124/jpet.114.218651 25277141

[77] Cartmell J, Monn JA, Schoepp DD. The metabotropic glutamate 2/3 receptor agonists LY354740 and LY379268 selectively attenuate phencyclidine versus d-amphetamine motor behaviors in rats. J Pharmacol Exp Ther. 1999; 291:161–170. 10490900

[78] Moreno JL, Holloway T, Albizu L, Sealfon SC, González-Maeso J. Metabotropic glutamate mGlu2 receptor is necessary for the pharmacological and behavioral effects induced by hallucinogenic 5-HT2A receptor agonists. J Neurosci Lett. 2011; 15:493:76–79. 10.1016/j.neulet.2011.01.046 PMC3064746 21276828

[79] Seeman P. An agonist at glutamate and dopamine D2 receptors, LY404039. Neuropharmacology 2013; 66: 87–8. 10.1016/j.neuropharm.2012.07.001 22884896

[80] Graham K. The more or less startling effects of weak prestimulation. Psychophysiology 1975; 12: 238–248. 1153628 10.1111/j.1469-8986.1975.tb01284.x

[81] Davis M, Gendelman DS, Tischler MD, Gendelman PM. A primary acoustic startle circuit: lesion and stimulation studies. J Neurosci.1982; 2:791–805. 7086484 10.1523/JNEUROSCI.02-06-00791.1982 PMC6564345

[82] Swerdlow NR, Geyer MA, Braff DL. Neural circuit regulation of prepulse inhibition of startle in the rat: current knowledge and future challenges. Psychopharmacology 2001; 156:194–215. 11549223 10.1007/s002130100799

[83] Koch M. 1999 The neurobiology of startle. Prog Neurobiol. 59; 107–128. 10463792 10.1016/s0301-0082(98)00098-7

[84] Tregellas J.R., Ellis J, Shatti S, Du Y.P., Rojas D.C.. Increased hippocampal, thalamic, and prefrontal hemodynamic response to an urban noise stimulus in schizophrenia. Am J Psychiatry 2009;166: 354–60. 10.1176/appi.ajp.2008.08030411 19147695 PMC2886660

[85] Grauer SM, Pulito VL, Navarra RL, Kelly MP, Kelley C, Graf R et al Phosphodiesterase 10A inhibitor activity in preclinical models of the positive, cognitive, and negative symptoms of schizophrenia. J Pharmacol Exp Ther. 2009; 331:574–90 10.1124/jpet.109.155994 19661377

[86] Gresack JE, Seymour PA, Schmidt CJ, Risbrough VB. Inhibition of phosphodiesterase 10A has differential effects on dopamine D1 and D2 receptor modulation of sensorimotor gating. Psychopharmacology. 2014; 231:2189–97. 10.1007/s00213-013-3371-7 24363077 PMC4017785

[87] Galici R, Echemendia NG, Rodriguez AL, Conn PJ. A selective allosteric potentiator of metabotropic glutamate (mGlu) 2 receptors has effects similar to an orthosteric mGlu2/3 receptor agonist in mouse models predictive of antipsychotic activity. J Pharmacol Exp Ther. 2005; 3153:1181–7. 10.1124/jpet.105.091074 16123306

[88] Cadenhead KS. Vulnerability markers in the schizophrenia spectrum: implications for phenomenology, genetics, and the identification of the schizophrenia prodrome. Psychiatr Clin North Am.2002; 25:837–53. 12462863 10.1016/s0193-953x(02)00021-7

[89] Schwarzkopf SB, Lamberti JS, Smith DA. Concurrent assessment of acoustic startle and auditoryP50evoked potential measures of sensory inhibition. Biol Psychiatry.1993; 33:815–28. 8373920 10.1016/0006-3223(93)90023-7

[90] Oranje B, Geyer MA, Bocker KB, Leon Kenemans J, Verbaten MN. Prepulse inhibition and P50 suppression: Commonalities and dissociations. Psychiatry Research 2006; 30: 147–158. 10.1016/j.psychres.2005.11.002 16879870

[91] Simosky JK, Stevens KE, Adler LE, Freedman R. Clozapine improves deficient inhibitory auditory processing in DBA/2 mice, via a nicotinic cholinergic mechanism. Psychopharmacology 2003; 165:386–396. 12459928 10.1007/s00213-002-1285-x

[92] Stevens KE, Wear KD. Normalizing effects of nicotine and a novel nicotinic agonist on hippocampal auditory gating in two animal models. Pharmacol Biochem Behav. 1997; 57:869–874. 9259018 10.1016/s0091-3057(96)00466-2

